# Unraveling the pathogenomics of *Rhizoctonia solani* infecting proso millet (*Panicum miliaceum* L.): genomic perspective on ruthless virulence and adaptive evolution

**DOI:** 10.3389/fmicb.2025.1557991

**Published:** 2025-03-07

**Authors:** Prasanna S. Koti, T. S. S. K. Patro, K. B. Palanna, B. Jeevan, Porapu Prasanth, G. V. Ramesh, N. Anuradha, Y. Sandhya Rani, Ungata Triveni, K. Lavanya Devi, T. Tharana Poonacha, Farooq Khan, Boda Praveen, M. Divya, D. Sabina Mary, V. Prasanna Kumari, T. E. Nagaraja, R. Madhusudhana, C. Tara Satyavathi

**Affiliations:** ^1^Department of Plant Biotechnology, University of Agricultural Sciences, GKVK, Bengaluru, India; ^2^Agricultural Research Station, Acharya N. G. Ranga Agricultural University, Vizianagaram, India; ^3^ICAR-AICRP on Small Millets, Project Coordinating (PC) Unit, University of Agricultural Sciences, GKVK, Bengaluru, India; ^4^Crop Protection Division, ICAR-National Rice Research Institute, Cuttack, India; ^5^Krishi Vigyan Kendra (KVK), Nadia II, Eastern Regional Station, ICAR-National Dairy Research Institute (NDRI), Kalyani, India; ^6^Functional Genomics and Bioinformatics Theme Group, The University of Trans-Disciplinary Health Sciences and Technology, Bengaluru, India; ^7^Department of Plant Pathology, University of Agricultural Sciences, GKVK, Bengaluru, India; ^8^Department of Plant Pathology, Agricultural College, Acharya N. G. Ranga Agricultural University, Bapatla, India; ^9^ICAR-Indian Institute of Millets Research, Hyderabad, India

**Keywords:** *Rhizoctonia solani*, proso millet, genetic diversity, CAZymes, secretome, retrotransposons

## Abstract

**Introduction:**

Banded sheath blight (Bsb), caused by *Rhizoctonia solani*, is an emerging threat to proso millet cultivation, significantly impacting yield and grain quality. This study on the pathogenomics of *R. solani* seeks to unravel its genetic mechanisms, identify key virulence factors, decode host-pathogen interactions, and pinpoint molecular targets for effective control strategies.

**Methods:**

*R. solani* isolates were collected from various regions across India, resulting in six distinct isolates. These isolates were comprehensively characterized through morphological observations, molecular analyses, and virulence assessments to gain comprehensive insights into their diversity and pathogenic potential. The most virulent strain, designated VAP-1, infecting proso millet, was sequenced using the Illumina platform and *de novo* assembled using the SPAdes assembler, resulting in a highly complete genome. Functional regions of the genome were predicted and annotated using Funannotate. A subsequent comparative genomics study and secretome analysis were conducted to support functional genomic investigations.

**Results:**

The VAP-1 genome assembly resulted in a total size of 47.12 Mb, with approximately 17.62% of the genome consisting of repetitive sequences, predominantly dominated by interspersed elements (around 97.8%). These interspersed elements were primarily classified as retrotransposons (72%), with DNA transposons comprising a smaller proportion (5%), while the remaining interspersed sequences were not fully annotated. Functional analysis of the genome revealed significant enrichment in KEGG pathways, including “Carbohydrate metabolism,” “Translation,” “Signal transduction,” and “Transport and catabolism.” In addition, Gene Ontology (GO) terms such as “Proteolysis,” “Membrane,” and “ATP binding” were notably enriched. The secretory protein profile of the VAP-1 genome from *R. solani* features key proteins from the major facilitator superfamily (MFS) transporters, (Trans) glycosidases, P-loop containing nucleoside triphosphate hydrolases, and galactose oxidase, all within the central domain superfamily. Glycoside hydrolases represent the largest class of CAZymes in the VAP-1 genome. Comparative genomic analysis of VAP-1 with other *R. solani* strains infecting Poaceae (e.g., rice) and non-Poaceae (e.g., sugar beet and tobacco) hosts showed that VAP-1 clusters closely with rice-infecting strains at the species level, yet exhibits a greater divergence in genomic similarity from strains infecting sugar beet and tobacco. Notably, variations were observed in important secretory proteins, such as multiple base deletions in MFS proteins across strains infecting proso millet, rice, and sugar beet.

**Discussion:**

Functional analysis of the VAP-1 genome has unveiled a wealth of insights, though we have only begun to scratch the surface. KEGG and GO annotations point to critical proteins that are essential for host infection, providing the pathogen with a potent arsenal for successful penetration, survival, and dissemination within the host. The secretory proteins encoded in the VAP-1 genome play a pivotal role in equipping the pathogen with the necessary tools to degrade plant cell wall polymers, release cell wall-bound saccharides, and break down polysaccharides for energy utilization and host colonization. Notable variations were observed in several secretome superfamily proteins within the VAP-1 strain. These findings underscore the genomic diversity present within *R. solani* strains and suggest possible adaptations that may contribute to host specificity.

## Introduction

Millets, ancient grains belonging to the Poaceae family, are often categorized as inferior coarse cereals. However, these grains are increasingly attracting global interest due to their superior nutritional profile and associated health benefits compared to more commonly consumed cereal staples. As a result, researchers are re-evaluating them as “super cereals.” Despite their potential, smallholder farmers, typically working with limited resources and minimal inputs, grow these grains primarily on marginal lands (Vetriventhan et al., 2020; Palanna et al., 2023a, 2023b). Among the minor millets, proso millet (*Panicum miliaceum* L.) stands out as an important crop, cultivated for food, feed, and fodder across Asia, particularly in India, Nepal, Pakistan, Myanmar, and Sri Lanka. Although small millets are known for their resilience to adverse environmental conditions, the emergence of banded sheath blight (Bsb), a disease caused by the pathogenic fungus *Rhizoctonia solani*, has become a major threat. This fungal infection has recently led to significant crop losses in many small millets, including proso millet (Patro et al., 2024).

The soil-borne pathogen *R. solani* (teleomorph: *Thanatephorus cucumeris*) is a notorious threat, known to infect over 100 plant species and potentially impacting a wide range of cultivated crops (Chahal et al., 2003; Ajayi-Oyetunde and Bradley, 2018; Patro et al., 2020). Isolates of *R. solani* are classified into 14 anastomosis groups (AG1 to AG13 and AGB1) based on their ability to undergo mycelial fusion, a trait that reflects their genetic similarities. These groups are further subdivided into 28 intergenic groups, characterized by differences in host range, pathogenicity, and genetic and biochemical properties (Carling et al., 2002; Singh et al., 2019). Given the global significance of *R. solani* and its intricate genetic diversity, extensive research is underway to explore the molecular mechanisms underlying its pathogenicity, employing a variety of omics approaches to deepen our understanding (Razali et al., 2021).

In nature, plants and pathogens engage in a constant battle, co-evolving as they adapt to one another’s strategies. Plants have developed sophisticated mechanisms to recognize and respond to pathogens, triggering defense systems that activate innate immunity to prevent invasion. The two primary defense mechanisms plants employ are PAMP-triggered immunity (PTI) and effector-triggered immunity (ETI) (Jones and Dangl, 2006). However, pathogens have countered these defenses by evolving the ability to overcome host resistance through the secretion of key pathogenic components known as effectors (Stergiopoulos and de Wit, 2009; Sunani et al., 2024). These effector proteins are typically cysteine-rich, an amino acid containing sulfur that forms disulfide bridges, thus stabilizing their tertiary structures and making them more resistant to degradation (Ciuffetti et al., 2010; Khang et al., 2010). It is hypothesized that conserved motifs within fungal effectors, such as the Y/F/WxC, LFLAKHVLVXXP, and RXLR motifs, play crucial roles in effector uptake, suggesting that these motifs have specialized functions in pathogenesis (Liu et al., 2024).

Moreover, carbohydrate-active enzymes (CAZymes) are vital pathogenic factors secreted by plant pathogenic fungi, enabling them to breach plant cell walls and gain access to host tissues (Rytioja et al., 2014). In a previous secretome analysis of *R. solani* (RAP2) infecting barnyard millet (*Echinochloa frumentacea*), the top three predicted CAZymes were glycoside hydrolases, auxiliary activities, and glycosyltransferases. Additionally, a considerable number of secreted proteins from the virulence factor or pectin lyase fold superfamily were identified, which hydrolyze plant cell wall polymers to release saccharides. Notably, a comparison of the cutinase protein domains of *R. solani* strains infecting rice, maize, and soybean revealed a 28-bp deletion in the cutinase gene of the RAP2 strain. This deletion may play a critical role in enabling the pathogen to infect barnyard millet, potentially allowing it to bypass the host’s defense mechanisms (Patro et al., 2024).

Thus, pan-genome analysis offers valuable insights into the molecular basis of pathogenesis, host adaptability, and genetic diversity within species by distinguishing core from accessory genes. This approach also aids in identifying key genes involved in pathogenicity, host adaptation, and potential drug targets. To investigate the genetic composition of *R. solani* infecting proso millet, the current study aims to: (1) analyze the virulence and characterize *R. solani* isolates responsible for banded sheath blight (Bsb) disease in proso millet, and (2) employ genome sequencing and comparative analysis to explore the similarities and differences between *R. solani* strains infecting various crops.

## Materials and methods

### Collection of disease samples, isolation, and purification of pathogen

During the *Kharif* season of 2021, disease samples were collected from proso millet plants exhibiting symptoms of banded sheath blight. These samples, comprising infected leaves and leaf sheaths, were gathered from the Indian states of Odisha, Chhattisgarh, and Andhra Pradesh, as part of a survey (Table 1).

**Table 1:** Details of isolates gathered from various proso millet-growing regions across India for this study.

Sl. No.	Isolate	Isolate code	Place of collection	State
1	Isolate-1	BOD-1	Brahmapur	Odisha
2	Isolate-2	SOD-2	Semiliguda	Odisha
3	Isolate-3	JCG-1	Jagdalpur	Chhattisgarh
4	Isolate-4	RCG-2	Raipur	Chhattisgarh
5	Isolate-5	VAP-1	Vizianagaram	Andhra Pradesh
6	Isolate-6	HAP-2	Hiramandalam	Andhra Pradesh

Proso millet leaves and leaf sheaths showing characteristic symptoms of banded sheath blight, collected from various locations were used for pathogen isolation on Potato Dextrose Agar (PDA) medium. Leaf bits measuring approximately 4 mm^2^, including both healthy and infected portions, were excised and surface sterilized using 1% sodium hypochlorite for 1 min. This was followed by three rinses with sterilized distilled water to ensure complete removal of the disinfectant. The sterilized leaf segments were then placed between sterile blotting sheets to remove excess moisture before being aseptically transferred to PDA plates (Ramesh et al., 2021; Sunani et al., 2025). The plates were incubated at 27 ± 1°C, and observations for mycelial growth were made at 24-h intervals. After 2 days, mycelial bits from the diseased leaf segments were aseptically transferred onto glass slides for microscopic examination. Morphological characteristics, including hyphal width, branching angle, and septation, were assessed to confirm the pathogen’s identity. The confirmed pathogen cultures were subsequently sub-cultured on PDA for further study (Singh et al., 2018).

### Pathogenicity and virulence analysis

The pathogenicity of the test isolates was assessed by inoculating one-month-old plants of the susceptible variety, Nilavoor local. Seedlings were cultivated in plastic pots measuring 30 cm in diameter and 20 cm in depth, containing a sterilized soil and vermicompost mixture in a 2:1 ratio. The leaf sheath was inoculated following the method outlined by Shekhar and Kumar (2012). Each pot contained two plants, with three replications maintained for each treatment. Control plants, which were not inoculated with the pathogen, were also included. Disease responses and the percent disease index were assessed 25 days’ post-inoculation using the standard assessment scale described by Palanna and Das (2022).

### Cultural and morphology analysis

Cultural characteristics were recorded when the hyphae reached the edge of the Petri dishes, focusing on observations of colony color, growth pattern, colony texture, and hyphal width. Morphological diversity among the isolates was assessed based on hyphal characteristics and sclerotial features, including their color, texture, size, and formation patterns, as well as the arrangement of sclerotia. Mycelial masses from a 10-day-old culture were mounted and photographed using an Olympus BX 51 microscope equipped with a Progres 2.7 (Jenoptik, United States) digital camera for microscopic examination (Ram et al., 2024). Sclerotial characteristics were recorded by analyzing an average of 25 sclerotia for each isolate.

### DNA extraction, PCR amplification and phylogenetic analysis

Cultures incubated for 5 days at 27 ± 1°C on potato dextrose broth (PDB) and inoculated with mycelial discs were used for DNA extraction following the CTAB method (Murray and Thompson, 1980). DNA concentration was measured using a Nanodrop spectrophotometer (JENWAY Genova Nano Ver.1.58.3) at the 260/280 nm spectrum. The ITS region of rDNA from all six isolates was amplified using the primer pair ITS-1 (5′-TCCGTAGGTGAACCTGCGG-3′) and ITS-4 (5′-TCCTCCGCTTATTGATATGC-3′) with an annealing temperature of 57°C (Patro et al., 2024). The amplified PCR products were sequenced. The obtained sequences were aligned using Clustal W. Additional ITS-5.8S rDNA sequences of *R. solani* isolates representing different anastomosis groups from the NCBI GenBank were included for phylogenetic analysis. A phylogram illustrating the relationships among *R. solani* isolates was constructed using the neighbor-joining method (Patil et al., 2024; Sandilya et al., 2022).

### Genome sequencing, assembly, gene prediction, and annotation

DNA libraries were prepared using the TruSeq DNA Nano LP kit and quantified with a Qubit 4 Fluorometer. Sequencing was performed on the Illumina NovaSeq 6,000 platform, generating 154 bp paired-end reads. The quality of the raw reads was assessed using FastQC v. 0.11.9 (Andrews, 2019). Subsequently, adapter sequences were removed, and quality filtering was applied using default parameters. Additionally, the first 10 bases of the 5′ end of each paired-end read were trimmed using Trim Galore v.0.6.5 (Felix, 2022).

The adapter-free, quality-filtered reads were assembled *de novo* with default settings using SPAdes v.3.15.5 (Bankevich et al., 2012). Genome-guided assembly correction and scaffolding were performed on the finished assembly using the *R. solani* AG-1 IA strain RefSeq genome (GCF_016906535.1) as a reference, with RagTag v.2.1.0 (Alonge et al., 2022). To eliminate repetitive contigs, the assembly scaffold was further refined using Funannotate v. 1.8.7 (Palmer and Stajich, 2020), with contigs shorter than 500 bp removed to generate the final genome. The adapter-free, quality-filtered reads were then mapped back to the finished genome using the bwa-mem v.0.7.18 (Heng, 2013) aligner with default settings, to evaluate the read utilization in the assembly process. Genome assembly quality was assessed using QUAST v.5.2.0 (Gurevich et al., 2013), and genome completeness was evaluated with CEGMA (Parra et al., 2007) on the gVolante (Nishimura et al., 2017) web server using the default settings and the CEGMA eukaryotic ortholog set for non-vertebrate gene prediction.

Gene prediction was performed using the “predict” command of the Funannotate v.1.8.7 pipeline, with RNA sequences from the *R. solani* AG-1 IA strain RefSeq (GCF_016906535.1) genome used as transcript evidence. Predicted protein sequences were annotated by creating a local BLAST database of UniProt proteins (The UniProt Consortium, 2023), followed by homology searches using Protein BLAST (Altschul et al., 1990; Camacho et al., 2008).

### Repetitive genomic elements of the VAP-1 genome

The repetitive landscape of the VAP-1 genome was characterized through the identification and annotation of repetitive elements. *De novo* prediction of VAP-1 specific repetitive sequences was performed using RepeatModeler v.2.0.3 (Flynn et al., 2020), and sequences with hits to known elements were annotated using the Repbase release v.20181026 (Bao et al., 2015). RepeatMasker v.4.1.3-p2 (Tarailo-Graovac and Chen, 2009) was employed to mask and annotate simple repeats, curated fungal repeats from the Repbase database, and genome-specific repeats. Additionally, the VAP-1 genome was scanned for simple sequence repeats, which were classified using the MegaSSR online server (Morad et al., 2023).

### Pathway and Gene Ontology analysis

The protein sequences of the VAP-1 assembly were subjected to analysis using BlastKOALA (Kanehisa et al., 2016) to determine their Kegg Orthology (KO) identifiers. Pathway annotations were then carried out through KEGG Mapper (Kanehisa and Sato, 2020; Kanehisa et al., 2022). To further investigate the functional implications, the UniprotR package v.2.3.0 (Soudy et al., 2020) was employed to query Gene Ontology (GO) terms, identifying the top 10 enriched terms associated with biological processes, molecular functions, and subcellular localization.

### Secretome analysis

To predict the fungal secretome of the VAP-1 genome, a standardized workflow was established, mirroring the methodology used in a previous study for the *in silico* secretome prediction of the ascomycete fungus *Parascedosporium putredinis* NO1 (Scott et al., 2023). For subcellular localization prediction, the protein sequences of the VAP-1 genome were input into the DeepLoc v. 2.1 server (Ødum et al., 2024). Subsequently, secretion prediction was performed using the SignalP v. 6.0 (Teufel et al., 2022) and TargetP v. 2.0 (Armenteros et al., 2019) servers. The resulting localization and secretory protein sequences were then cross-referenced to remove redundancies, retaining only unique proteins. These unique proteins were further analyzed using DeepTMHMM v. 1.0 (Hallgren et al., 2022) to identify potential transmembrane proteins. Among these, proteins predicted to possess alpha-helical or beta-barrel structures as transmembrane proteins were considered as part of the secretome of the VAP-1 genome. Superfamily annotations were derived by screening the secretome proteins against the InterPro protein signature databases (Paysan-Lafosse et al., 2022). Carbohydrate-active enzymes (CAZymes) in the VAP-1 genome were annotated using the dbCAN3 web server (Jinfang et al., 2023), employing the HMMER tool for CAZyme family identification through a search against the dbCAN CAZyme domain HMM database. Additionally, CAZyme subfamily annotations were obtained via an HMMER search against the dbCAN-sub HMM database, which categorizes CAZyme subfamilies.

### Comparative genomics analysis

The genome of the highly virulent VAP-1 isolate of *R. solani* from proso millet, which belongs to the Poaceae family, was compared to the genomes of other Poaceae species, such as rice. Additionally, to explore broader genetic relationships, the VAP-1 genome was also compared with those of *R. solani* strains infecting non-Poaceae plants, including sugar beet and tobacco. The genome sequences for *R. solani* strains infecting rice (GCA_015342415.1), sugar beet (GCA_905219575.1), and tobacco (GCA_905219615.1) were retrieved from the NCBI Genomes database (Sayers et al., 2021). These genomic datasets were uploaded to the MOSGA web server (Martin et al., 2020, 2021) to assess genome completeness and to calculate the average nucleotide identity (ANI) between the species. To further investigate functional similarities, the protein sequences of the four *R. solani* genomes were analyzed through the OrthoVenn3 web server (Sun et al., 2023), which identified orthologous gene clusters and provided insights into their functional relevance across the genomes.

### Variation and evolution of secretome proteins

The secretome protein repertoire of *R. solani* (VAP-1), comprising 124 proteins, was analyzed with *R. solani* genome’s proteins from rice, sugar beet, and tobacco. This analysis was conducted through a homologous search using BLASTP v.2.10.0+ with the 124 secretome proteins of *R. solani* (VAP-1) as queries against the complete protein sequences of *R. solani* from rice, sugar beet, and tobacco. Homologous sequences with more than 70% identity were retained for further analysis. The homologous secretome proteins from *R. solani* infecting proso millet (VAP-1), along with those from rice, sugar beet, and tobacco, were submitted individually to the OrthoVenn3 web server (Sun et al., 2023) for gene family contraction and expansion analysis using CAFE (Mendes et al., 2020). A comparative analysis of the secretome proteins annotated with InterPro superfamily signatures, along with their homologous proteins from *R. solani* genomes infecting rice, sugar beet, and tobacco, was performed. This analysis focused on identifying variations in the conserved sequences of the proteins. Multiple sequence alignments of Major Facilitator Superfamily (MFS), transglycosidases, P-loop containing nucleoside triphosphate hydrolases, and galactose oxidases were conducted using the Clustal Omega web server (Sievers and Higgins, 2018).

## Results

### Disease symptoms, and pathogen isolation

**Figure 1:**
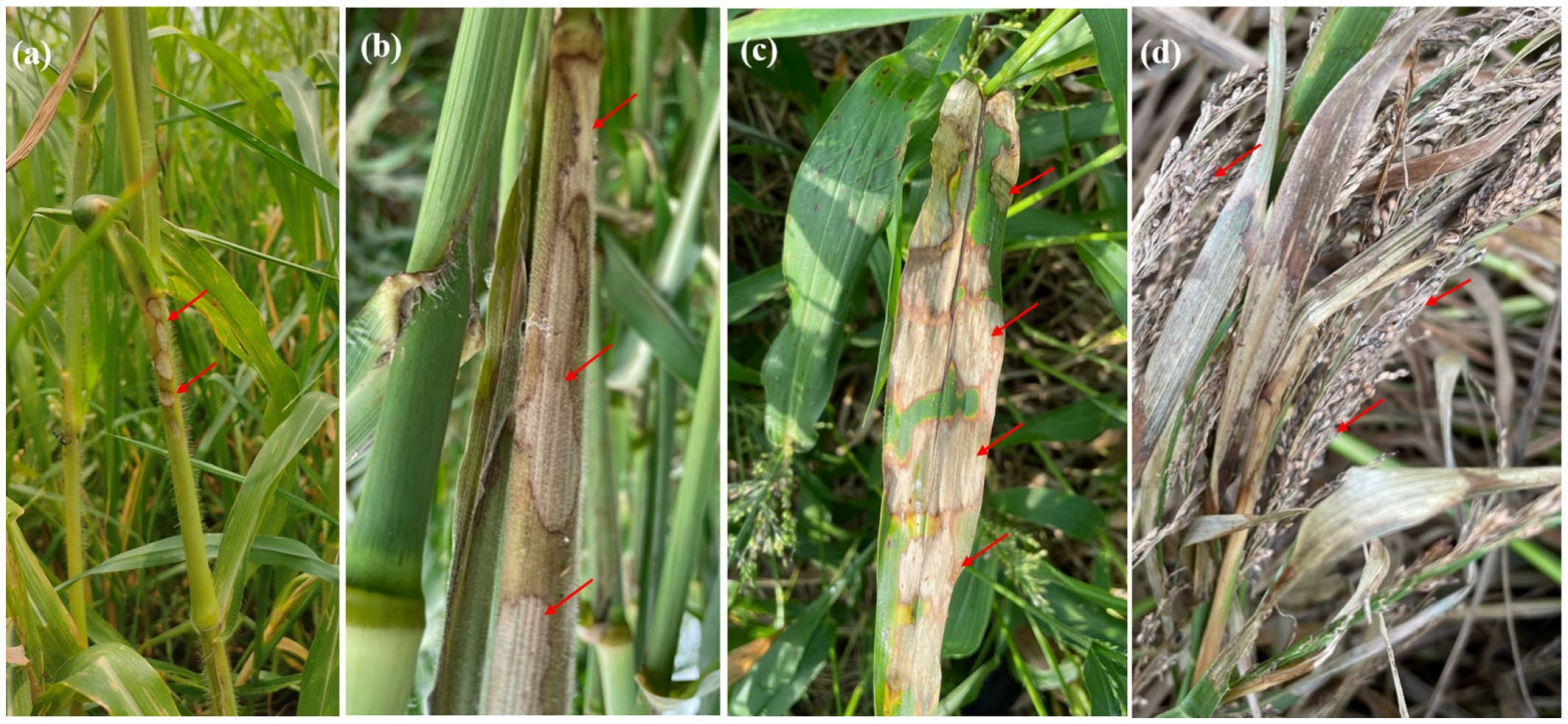
Symptoms of banded sheath blight on proso millet include: **(a)** initial appearance of small lesions on the leaf sheath. **(b)** Development of elongated, oval-shaped lesions on the stem. **(c)** A characteristic banded appearance on the leaves, marked by straw-colored lesions with distinct brown margins. **(d)** Advanced stages affecting the panicle, leading to grain discoloration and drying.

Bsb symptoms initially appear as small, irregularly shaped white lesions on the lower leaf sheath, located between the soil line and the leaf blade. Over time, these lesions expand to the upper leaf sheaths, forming 2–3 cm long oval to irregular lesions that transition from light grey to dark brown. Eventually, the lesion centers become greyish white to straw-colored, bordered by narrow reddish-brown margins. These margins form a series of brown lines along the leaf sheaths, creating a distinct banded appearance. As the disease progresses, the lesions merge, enlarge, and extend upward, ultimately causing blighting of the leaf sheath and leaf blade (Figure 1). Bsb diseases samples were obtained from infected proso millet plants across various regions of Indian states and were purified using the hyphal tip method, following standard isolation techniques. Six *R. solani* isolates were recovered, each assigned a unique identity code (Table 1).

### Morphology and cultural characteristics

**Figure 2:**
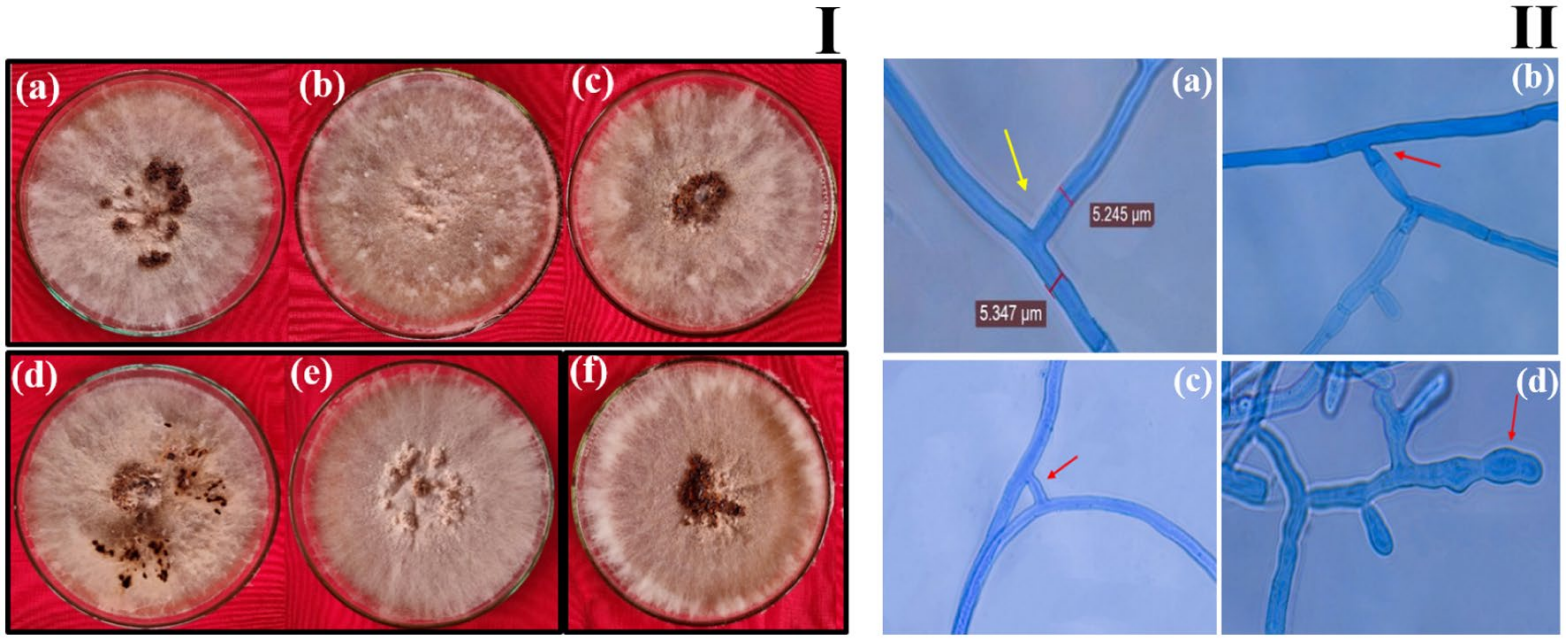
Morphological characteristics of *R. solani* isolates. **(I)** Colony morphology of *R. solani* isolates **(a)** BOD-1, **(b)** SOD-2, **(c)** JCG-1 **(d)** RCG-2 **(e)** VAP-1 **(f)** HAP-2. **(II)** Microscopic images (400×) depicting the hyphal characteristics of *R. solani*. **(a)** Right angle branching. **(b)** Constriction at point of origin. **(c)** Hyphal fusion. **(d)** Barrel shaped monilioid cells.

On PDA medium, the fungus formed yellow, cottony, fluffy colonies with both aerial and surface mycelia. The hyphal width ranged from 5.02 ± 0.20 μm to 7.42 ± 0.17 μm. Significant variation was observed among the isolates in their sclerotial characteristics. Sclerotia were formed either aerially or on the surface, with their color ranging from light brown to dark brown. The sclerotial diameter varied from 0.93 ± 0.05 mm to 1.79 ± 0.01 mm. The texture of the sclerotia was either rough or smooth, and their distribution was central, sub-central, or in a peripheral ring. Among the isolates, RCG-02 produced the highest number of sclerotia (32), while JCG-01 produced the fewest (13) (Supplementary Table 1) (see Figure 2).

### Pathogenicity and virulence analysis

The PDA plugs, containing actively growing mycelium of different isolates of *R. solani*, were inoculated on susceptible proso millet cultivar, Nilavoor local. All six isolates produced typical symptoms on leaf sheath 4 to 8 days’ post-inoculation. Using a standard scale (Palanna and Das, 2022), the virulence of the isolates was ascertained based on the percentage disease index (PDI). Nilavoor local showed typical lesions with ranged values of PDI from 22.60 to 65.50%. VAP-1 was the most virulent among the isolates, with a disease incidence of 65.65%, followed by HAP-2 (58.70%), RCG-2 (58.50%), BOD-1 (47.75%), and SOD-2 (41.54%), while JCG-1 had the lowest incidence of disease (22.60%).

### Molecular analysis to confirm the pathogen

**Figure 3:**
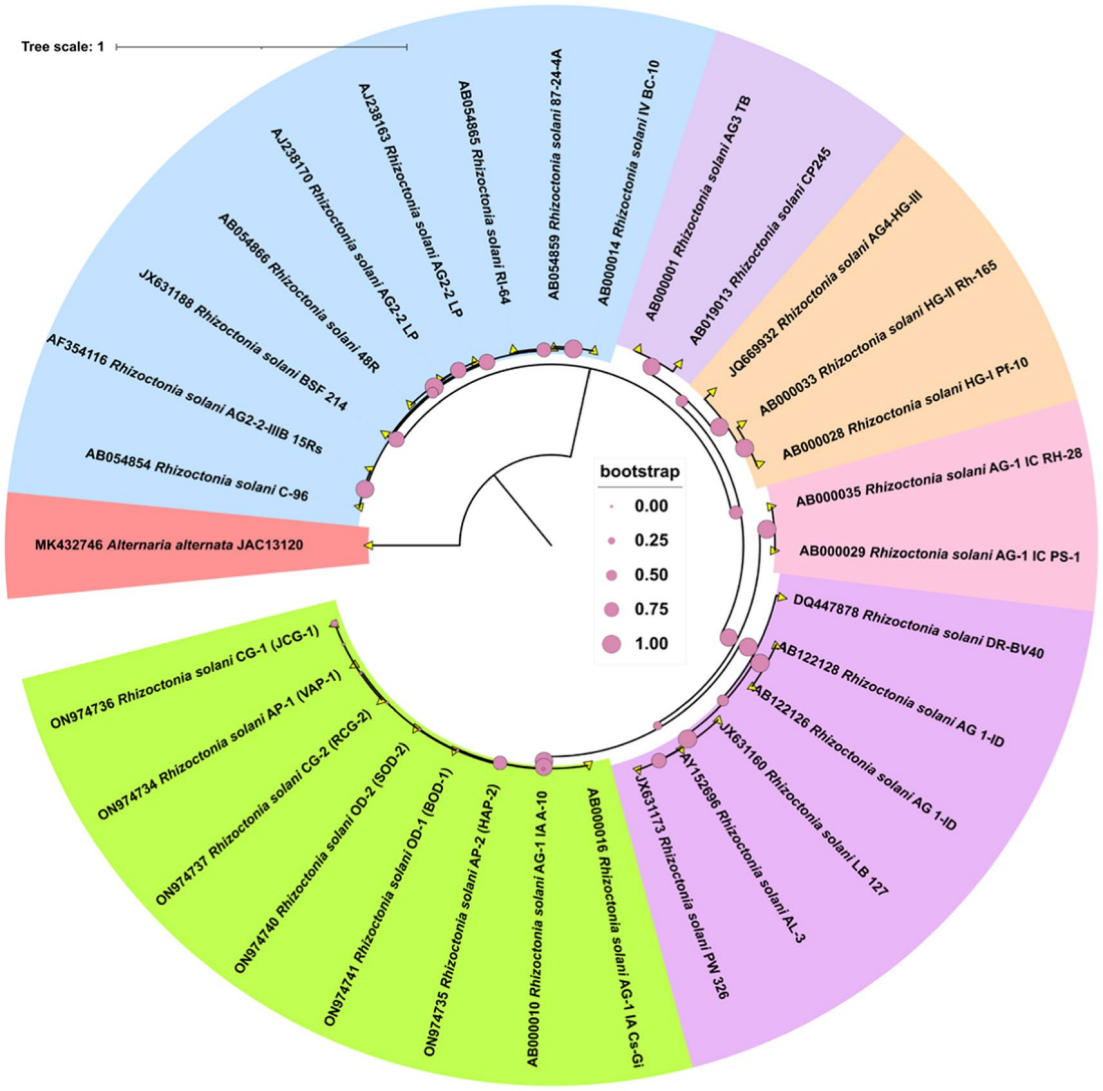
The phylogram generated from ITS sequences using the neighbor-joining method illustrates the genetic relationships among *R. solani* isolates. Bootstrap values, calculated from 1,000 replicates to produce a majority consensus tree, are indicated on the branches. The strain *Alternaria alternata* (JAC13120) was used as an outgroup for the analysis.

PCR amplification of the ITS region resulted in a fragment size of approximately 600 bp in length. The gene fragments were purified, and sequenced. Further, the gene sequences were submitted at NCBI GenBank. A phylogenetic tree was generated using the different gene sequences with various AGs and the ITS sequences from six *R. solani* isolates. All the six isolates showed highest similarity per cent with the AG-1 1A group of *R. solani* which confirms that all the six isolates belong to the anastomosis group, AG-1 1A subgroup (Figure 3).

### Genome sequencing, assembly, gene prediction, and functional annotation

**Figure 4:**
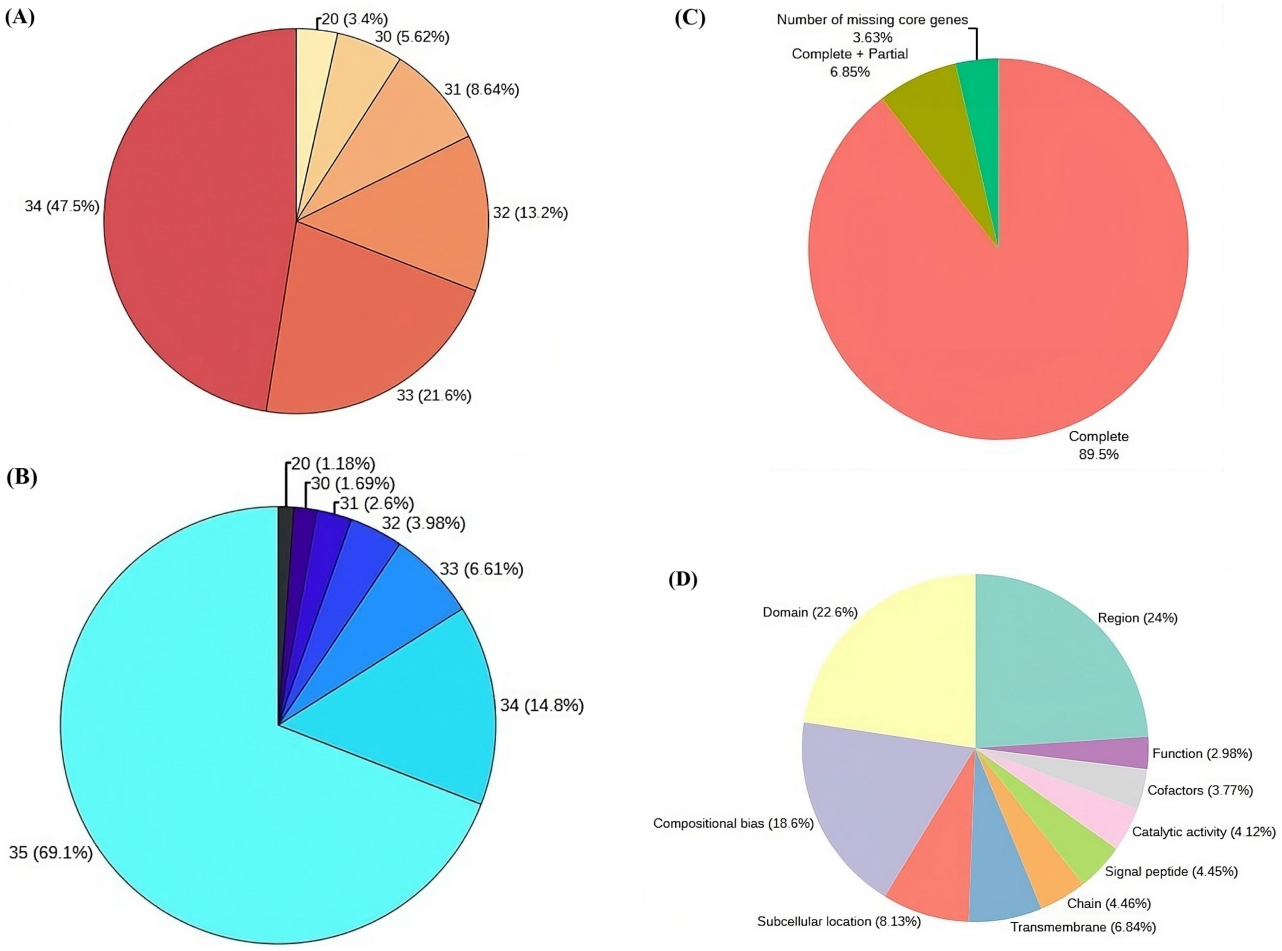
Genome sequencing, assembly, and protein annotation statistics. **(A)** Raw read statistics, with each segment annotated by Phred score and the corresponding percentage of reads in the library. **(B)** Quality-filtered read statistics, including Phred scores and the percentage of reads in the library. **(C)** CEGMA genome assembly completeness statistics **(D)** Top 10 sequence annotation features of proteins associated with UniProt.

Post virulence studies, the VAP-1 strain, identified as the most virulent, was selected for whole genome sequencing using the Illumina platform. The raw sequencing data consisted of 7.2 million 154 bp paired-end reads (Supplementary Table 2), with 96% of the reads having a Phred score greater than 30 (Figure 4A). After quality filtering, the library retained approximately 99.98% of the initial reads (Supplementary Table 2), with 98% of the reads having a Phred score greater than 30 (Figure 4B).

Following quality filtration, the *de novo* assembly of the reads resulted in a genome comprising 6,096 contigs, totaling approximately 47.12 Mb. Read utilization analysis indicated that 99.55% of the quality-filtered reads successfully mapped back to this assembled genome. The largest contig accounted for 8.3% of the total genome length, with N50 and N90 contig lengths of 2,122,470 bp and 2,398 bp, respectively. Additionally, the assembly’s L50 and L90 values were 8 and 1,207, respectively (Supplementary Table 3). When the assembly was evaluated against the eukaryotic ortholog dataset of 248 core non-vertebrate genes, it demonstrated 89.5% completeness, with 6.85% of genes partially complete and 3.63% missing (Figure 4C).

Gene prediction of the VAP-1 genome identified 9,321 protein sequences, which were subsequently annotated by comparing them to the 22,082 *R. solani* species-specific proteins obtained from the UniProt database. A homology search using BLASTP v.2.10.0+ revealed that approximately 81% of the 9,321 proteins exhibited more than 90% homology with *R. solani* specific protein sequences (Supplementary Table 4). Further analysis of the VAP-1 genome’s protein sequences annotations showed that the top three UniProt sequence annotation features of the sequences were *region*, *domain*, and *compositional bias*, comprising 24, 22.6, and 18.6% of the proteins, respectively (Figure 4D).

### Repetitive genomic elements of the VAP-1 genome

**Figure 5:**
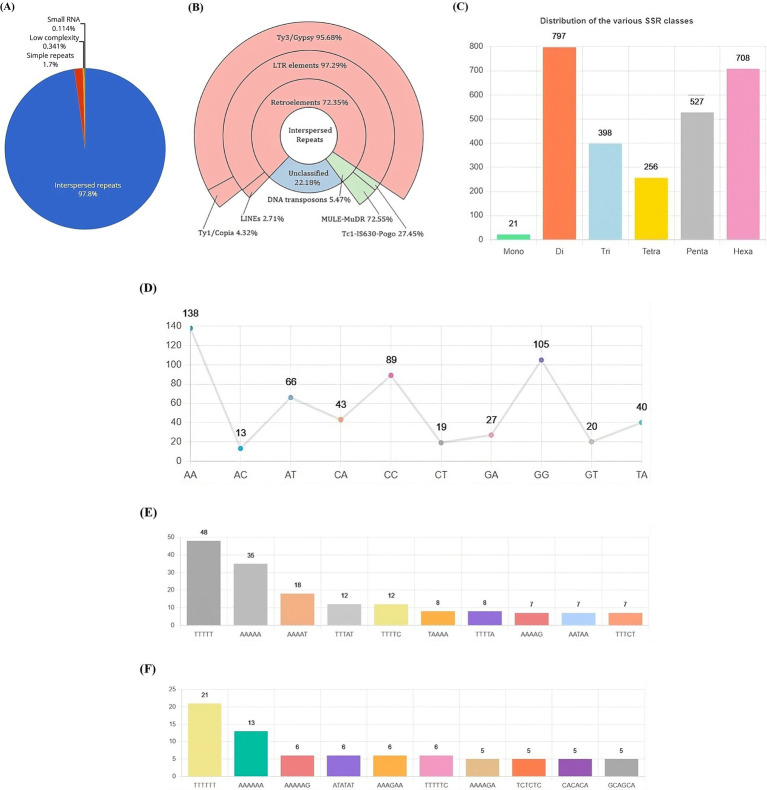
Repetitive DNA landscape of the *R. solani* VAP-1 isolate genome. **(A)** Composition of repetitive DNA. **(B)** Classification of interspersed repeats. **(C)** Distribution of SSR classes. **(D)** Proportion of di-nucleotide SSRs. **(E,F)** Distribution of top 10 penta-and hexa-nucleotide SSRs.

The repetitive landscape of the VAP-1 genome constitutes approximately 17.62% of its total sequence, with 97.8% composed of interspersed repeats, followed by minor contributions from simple repeats, low-complexity regions, and small RNA elements (Figure 5A). Interspersed repeats are predominantly composed of retroelements (72.35%), DNA transposons (5.47%), and unclassified repeats (22.18%). The retroelements are largely made up of LTR elements (97.29%) and LINEs (2.71%). Among the LTR elements, Ty3-retrotransposons account for 95.68%, while Ty1/Copia elements represent 4.32%. The DNA transposons are primarily composed of MULE-MuDR (72.55%) and Tc1-IS630-Pogo (27.45%) (Figure 5B). The most common simple sequence repeat (SSR) classes in the VAP-1 genome are di-, penta-, and hexanucleotide repeats (Figure 5C). The dominant dinucleotide SSRs are “AA” and “GG” (Figure 5D), while the penta-and hexanucleotide SSRs are “TTTTT” (Figure 5E) and “TTTTTT” (Figure 5F).

### Pathway and Gene Ontology analysis

**Figure 6:**
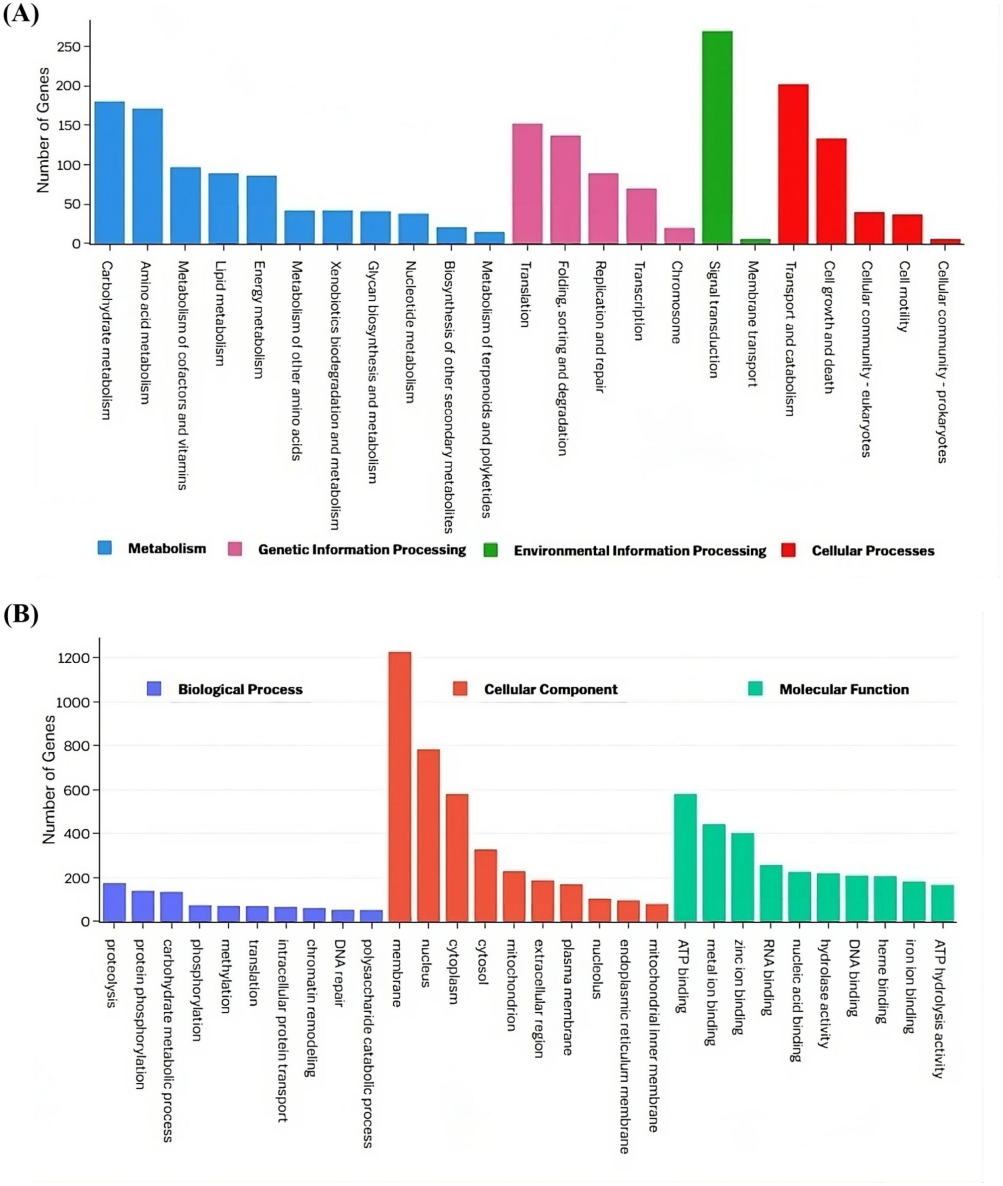
Functional annotation of the VAP-1 genome proteins infecting proso millet. **(A)** The KEGG annotation chart, with legends indicating the Level 1 hierarchy, where the bars represent the number of genes involved in the Level 2 pathways that fall under each Level 1 category. **(B)** The GO functional annotation classification statistics plot, where the *y*-axis represents the number of genes, and the *x*-axis displays a set of ontology terms corresponding to the Gene Ontology domains: molecular function, cellular components, and biological process.

The top three enriched KEGG pathway maps in the “Metabolism” category were “Carbohydrate metabolism,” “Amino acid metabolism,” and “Metabolism of cofactors and vitamins.” In the “Genetic Information Processing” category, the most enriched pathways included “Translation,” “Folding, sorting, and degradation,” and “Replication and repair.” The “Environmental Information Processing” category was dominated by the “Signal transduction” and “Membrane transport” pathways, while the “Cellular processes” category showed enrichment in “Transport and catabolism,” “Cell growth and death,” and “Cellular community-eukaryotes” (Figure 6A). Gene Ontology analysis revealed that the terms “proteolysis,” “membrane,” and “ATP binding” exhibited the highest gene enrichment within the “biological process,” “cellular component,” and “molecular function” domains, respectively (Figure 6B).

### Secretome analysis

**Figure 7:**
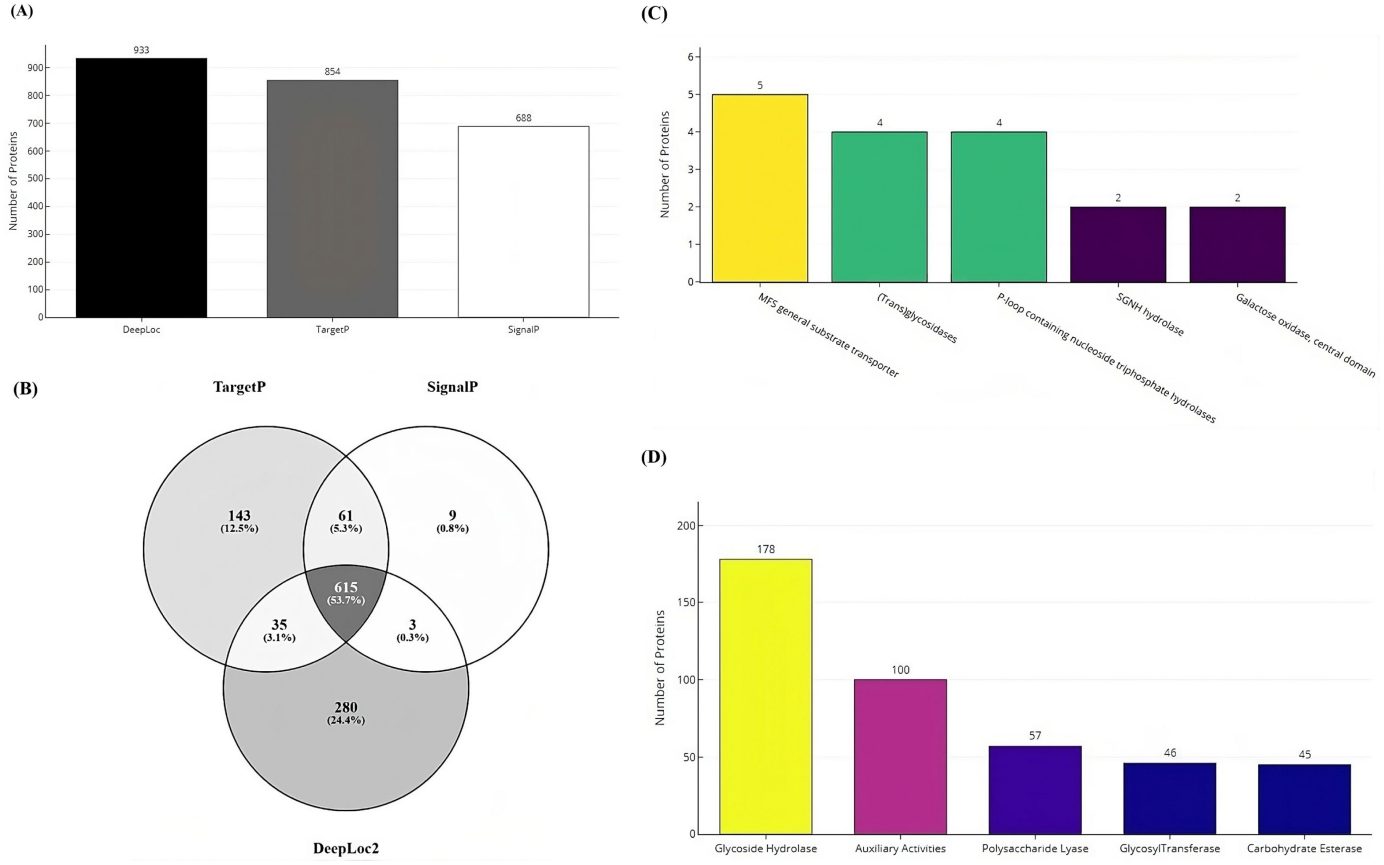
Secretome and CAZyme analysis of the VAP-1 genome. **(A)** Number of localization and secretory proteins predicted by the DeepLoc, TargetP, and SignalP tools. **(B)** Number of proteins shared across the DeepLoc, TargetP, and SignalP predictions. **(C)** SUPERFAMILY annotations of secretome proteins **(D)** Distribution of CAZyme classes predicted in the VAP-1 genome.

Secretome prediction began with the identification of subcellular localization and secretion proteins, resulting in the prediction of 933 proteins as localization proteins, and 854 and 688 proteins as secretory by the TargetP and SignalP tools, respectively (Figure 7A). The majority of these proteins (53%) were predicted to be both localization and secretory proteins (Figure 7B). Subsequently, 1,146 unique proteins were analyzed for the presence of alpha-helical or beta-barrel structures. Of these, 124 proteins were identified by DeepTMHMM as transmembrane proteins, which were then classified as part of the secretome of the VAP-1 genome. Further analysis using the EffectorP tool predicted 20 of these secretome proteins to contain signal peptides indicative of secretion. Superfamily annotations of the secretome proteins revealed that the top five categories included the MFS general substrate transporter, (Trans) glycosidases, P-loop containing nucleoside triphosphate hydrolases, SGNH hydrolase families and Galactose oxidase, central domain (Figure 7C). CAZyme classification identified the top five predicted enzymes as belonging to the Glycoside Hydrolases class, followed by Auxiliary Activities, Polysaccharide Lyases, Glycosyltransferases, and Carbohydrate Esterases classes (Figure 7D).

### Comparative genomics analysis

**Figure 8:**
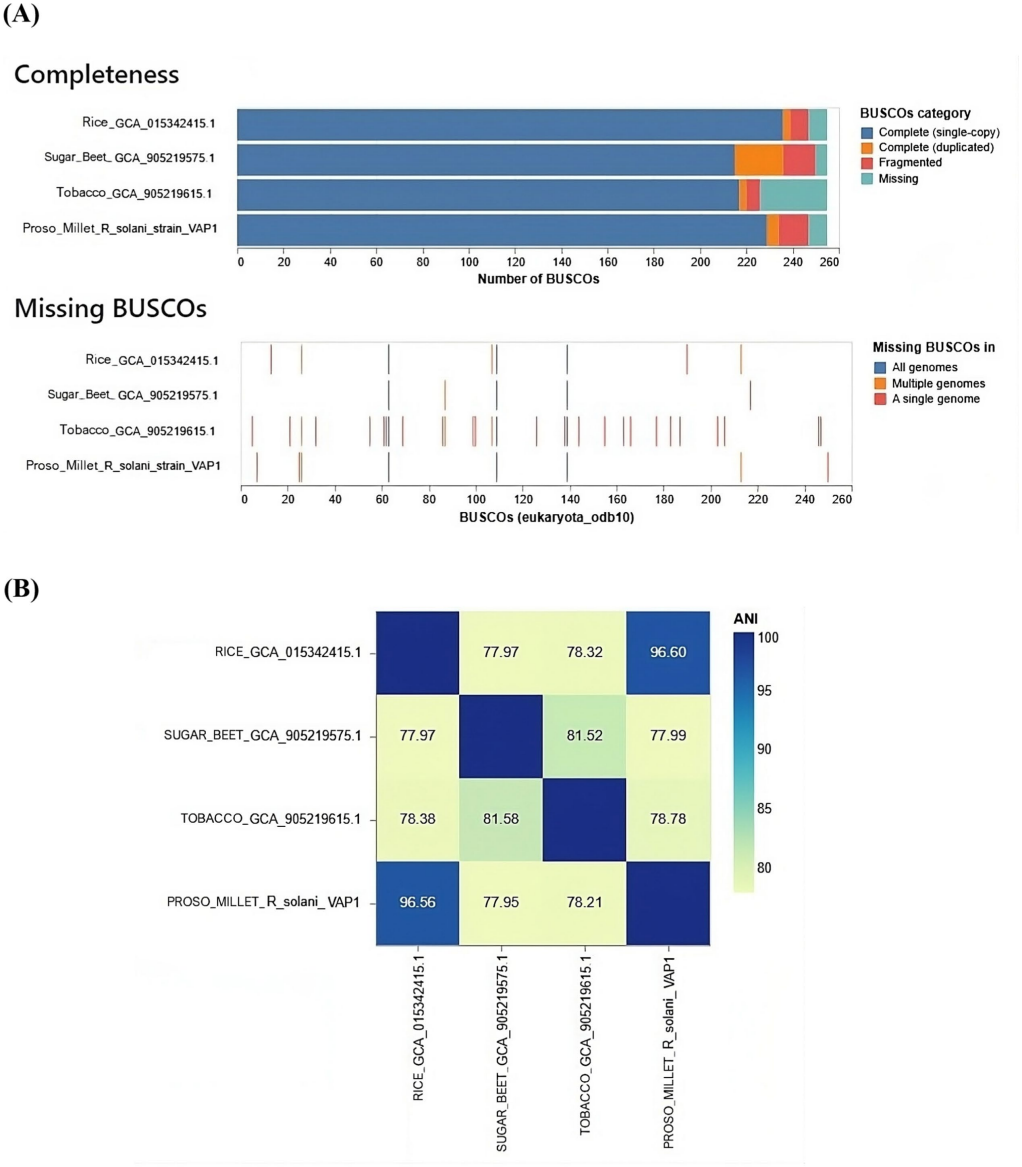
Comparative genomic analysis of *R. solani* genomes. **(A)** The assembly completeness of the *R. solani* genomes evaluated using the Eukaryota dataset of 260 BUSCOs, with the analysis quantifying both complete and missing BUSCOs. **(B)** The average nucleotide identity (ANI) as a measure of the genomic similarity among the *R. solani* genomes.

The gene content analysis revealed that the VAP-1 *R. solani* genome contained over 80% of complete BUSCOs from the 260 BUSCOs in the Eukaryota dataset. This was followed by a small proportion of fragmented and duplicated BUSCOs, with approximately 10% of BUSCOs missing. Among the genomes compared, the *R. solani* genome isolated from rice exhibited slightly superior BUSCO completeness compared to the VAP-1 genome, while *R. solani* genomes from sugar beet and tobacco showed fewer complete BUSCOs than VAP-1. In the comparison with the 260 BUSCOs from the Eukaryota dataset, the VAP-1 genome was found to be missing three orthologs, which were absent in all other *R. solani* genomes. Notably, the VAP-1 genome lacked one BUSCO, also absent in the tobacco and rice *R. solani* genomes, and another BUSCO that was missing in both VAP-1 and rice genomes. Three specific BUSCOs were missing only from the VAP-1 genome (Figure 8A).

The VAP-1 genome infecting proso millet and the *R. solani* genome infecting rice exhibited ANI values greater than 96%, suggesting a close species-level relationship. In contrast, the ANI values between the VAP-1 genome and the *R. solani* genomes infecting sugar beet and tobacco were below 96%, indicating a greater degree of genomic divergence (Figure 8B).

**Figure 9:**
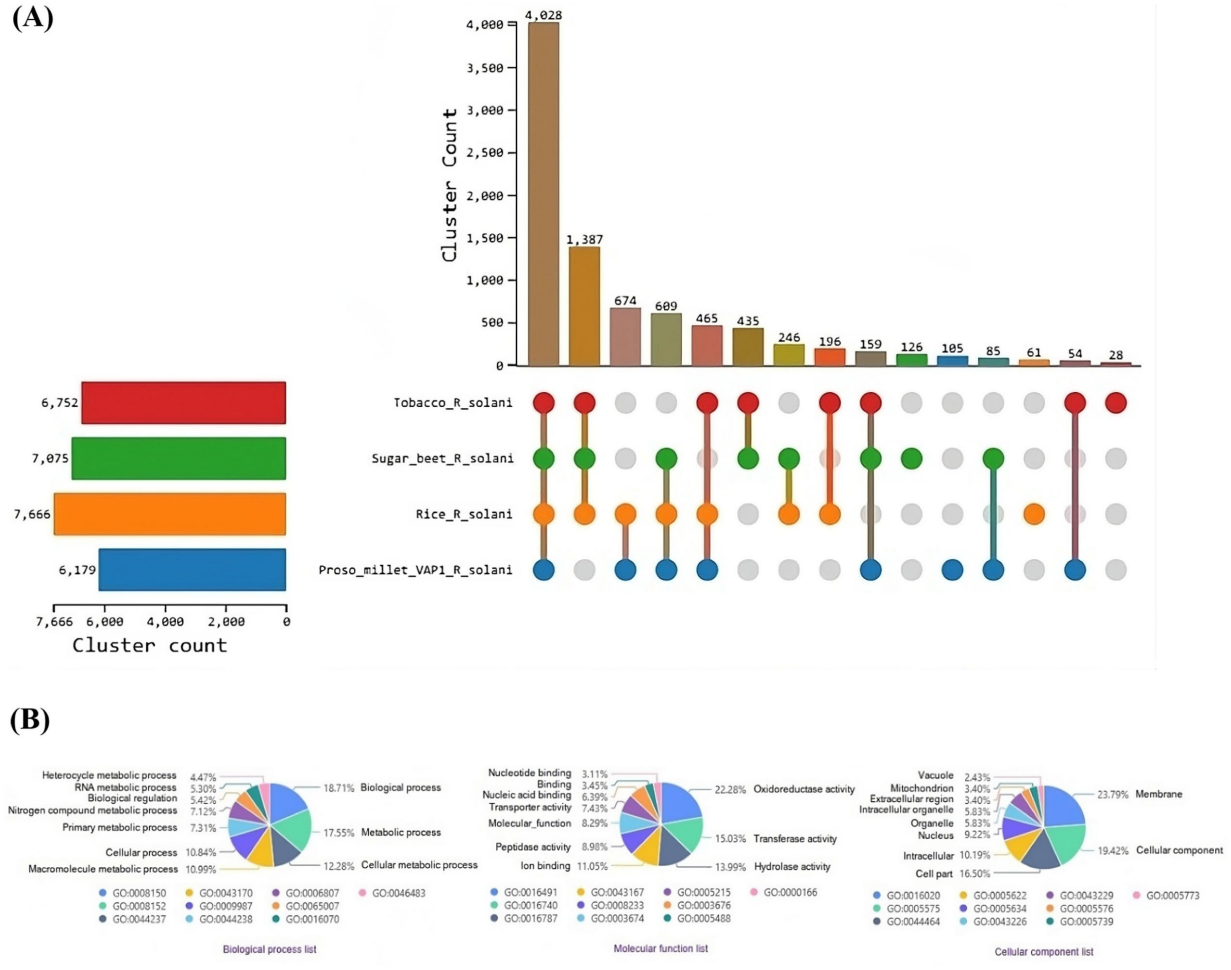
Comparative genomic analysis of protein sequences from *R. solani* genomes. **(A)** UpSet plot illustrating the distribution of orthologous protein clusters across the different *R. solani* genomes. **(B)** Enriched Gene Ontology (GO) terms of the shared orthologous protein clusters present in the *R. solani* genomes.

OrthoVenn3 analysis of VAP-1 protein sequences, compared with those from *R. solani* genomes infecting rice, sugar beet, and tobacco, identified 11 shared homologous gene clusters and 4 unique clusters across all species. A single large homologous gene cluster containing 4,028 proteins was common to all fungal genomes, while a unique cluster of 105 proteins was specific to the VAP-1 genome (Figure 9A). Functional Gene Ontology (GO) analysis of the 4,028 shared proteins revealed that the top three GO terms in the Biological Process domain were GO:0008150 (Biological process), GO:0008152 (Metabolic process), and GO:0044237 (Cellular metabolic process). In the Molecular Function domain, the top three GO terms were GO:0016491 (Oxidoreductase activity), GO:0016740 (Transferase activity), and GO:0016787 (Hydrolase activity). The top three GO terms in the Cellular Component domain were GO:0016020 (Membrane), GO:0005575 (Cellular component), and GO:0044464 (Cell part) (Figure 9B). The unique cluster of 105 proteins specific to the VAP-1 genome was enriched with five significantly associated GO terms, all within the Biological Process domain. These terms included GO:0033609 (oxalate metabolic process), GO:0009405 (pathogenesis), GO:0000272 (polysaccharide catabolic process), GO:0016052 (carbohydrate catabolic process), and GO:0009853 (photorespiration) (Table 2).

**Table 2:** OrthoVenn3 GO enrichment analysis of proteins clustered specifically to VAP-1 genome.

GO ID	GO Ontology	Name	Count	*p*-value
GO:0033609	Biological process	Oxalate metabolic process	7	0.0006
GO:0009405	Biological process	Pathogenesis	4	0.0072
GO:0000272	Biological process	Polysaccharide catabolic process	3	0.0005
GO:0016052	Biological process	Carbohydrate catabolic process	3	0.0099
GO:0009853	Biological process	Photorespiration	2	0.0016

### Variation and evolution of secretome proteins

Orthologous analysis of the secretome protein sequences from *R. solani* infecting proso millet, rice, sugar beet, and tobacco revealed that the secretomes of *R. solani* infecting sugar beet and tobacco have diverged into a distinct lineage, whereas the secretomes of *R. solani* infecting proso millet and rice form a separate, closely related lineage. Expansion and contraction analysis indicated that two gene families have contracted in *R. solani* infecting rice, while a single gene family has contracted in *R. solani* infecting sugar beet (Supplementary Figure S1A). A major facilitator superfamily protein, characterized as a transmembrane 9 superfamily protein from the VAP-1 genome, which shares greater than 70% identity with proteins from *R. solani* genomes of rice, sugar beet, and tobacco, was analyzed for variations. Multiple sequence alignment of these proteins revealed the presence of variable numbers of base deletions in *R. solani* infecting proso millet, rice, and sugar beet at different locations when compared to the tobacco isolate (Supplementary Figure S1B). Similarly, multiple sequence alignment of a single (Trans) glycosidases superfamily enzyme, specifically a Glycoside hydrolase family 10 protein, from *R. solani* infecting sugar beet, proso millet, and rice, revealed a single base mutation and consecutive two-base deletions in *R. solani* infecting proso millet and sugar beet when compared to rice isolate. The protein from *R. solani* infecting tobacco exhibited less than 70% identity and was therefore excluded from the analysis (Supplementary Figure S1C). Additionally, multiple sequence alignment of P-loop containing nucleoside triphosphate hydrolases, characterized as ATP-binding cassette subfamily B proteins from all *R. solani* genomes, revealed a 21-base deletion in *R. solani* infecting proso millet, located in a highly variable region surrounding the conserved region of the protein (Supplementary Figure S2A). Similarly, multiple sequence alignment of galactose oxidase proteins from *R. solani* infecting tobacco, proso millet, and rice revealed the presence of a 3-base deletion in the RAX2 domain in *R. solani* infecting tobacco and rice, compared to proso millet isolate (Supplementary Figure S2B).

## Discussion

The present study provides valuable insights into the pathological and molecular characteristics of *R. solani* isolates associated with Bsb in proso millet. Pathogenicity assays revealed significant variation in virulence among the *R. solani* isolates, with PDI values ranging from 22.60 to 65.50%. VAP-1 was identified as the most virulent isolate, with the highest disease incidence (65.65%), while JCG-1 exhibited the lowest virulence (22.60%). This variability underscores the significant intraspecific variability in fungal pathogens including *R. solani*, often associated to their complex genetic makeup and adaptability (Jeevan et al., 2023; Palanna et al., 2023a, 2023b). The findings emphasize the importance of region-specific screening of proso millet cultivars for resistance and the development of tailored fungicidal management strategies.

Molecular characterization further confirmed the identity of the isolates. PCR amplification, sequencing, and phylogenetic analysis of the ITS region identified all six isolates as belonging to the AG-1 1A subgroup of *R. solani*. This result aligns with previous studies indicating AG-1 1A as the primary anastomosis group associated with sheath blight in cereal crops (Liu et al., 2024; Patro et al., 2024). Given its high virulence, VAP-1 was selected for whole-genome sequencing to provide deeper insights into the genetic factors driving its pathogenicity.

In this study, we identified the most virulent *R. solani* strain (VAP-1) infecting proso millet and successfully sequenced, assembled, predicted, and annotated its functional genes. This work advances our understanding of the host’s genetic composition and offers valuable insights into the host-pathogen interactions between *R. solani* and proso millet. Sequencing on the Illumina platform generated high-quality paired-end reads, with 96% of the reads having a Phred score greater than 30. After applying quality filtration to remove adapter contamination, approximately 99.98% of the reads were retained for the genome assembly process. *De novo* assembly utilized 99.55% of the reads to assemble the VAP-1 genome, resulting in a total genome size of 47.12 Mb composed of 6,096 contigs. The largest contig is 3,918,178 bp in length, representing over 8% of the genome, while the N50 contig length is 2,122,470 bp, corresponding to the 8th largest contig (L50) in the assembly. The remaining 40% of the genome is composed of 1,199 contigs of varying sizes, with the N90 contig length measuring 2,398 bp.

The model reference strain (GCF_016906535.1) in the NCBI RefSeq database has a genome size of 40.85 megabases, consisting of 17 contigs. This is smaller compared to the VAP-1 genome in terms of both genome size and the number of contigs. The observed variation could be attributed to several factors, with the most significant being the repetitive content in phytopathogens, which may drive retrotransposon proliferation and genome size expansion (Spanu et al., 2010). Additionally, structural variations such as insertions, deletions, duplications, and inversions (Hartmann, 2021), as well as evolutionary adaptations to specific ecological niches (Naranjo-Ortiz and Gabaldón, 2019), may also contribute to the differences in genome characteristics.

Retrotransposons are known to play a critical role in fungal pathogenicity by promoting genome plasticity, regulating virulence factor expression, facilitating host adaptation, and conferring resistance to stress (Razali et al., 2019). In the VAP-1 genome, the majority of the repetitive content is composed of retrotransposons. Ty3-retrotransposons account for approximately 95% of the retrotransposons in the VAP-1 genome. These elements are known to enhance virulence during plant infection, and their derepression, followed by subsequent silencing, can lead to a reduction in virulence (Fouché et al., 2020).

ATP-driven efflux pumps, members of the ATP-binding cassette (ABC) superfamily, are critical for the transport of substrates across cellular membranes. Phytopathogenic fungi encounter toxic environments during infection as a result of plant defense responses. ABC transporters are essential for host infection, as they enable fungi to protect themselves against plant defense mechanisms (Urban et al., 1999). Gene Ontology (GO) annotations of proteins in the VAP-1 genome reveal that “ATP binding” is the most significantly enriched term in the “molecular function” domain. Within the “cellular component” domain, “membrane” is the most enriched category for VAP-1 proteins. In pathogenic filamentous fungi, the curvature of the cytoplasmic cell membrane is a contributing factor to fungal virulence (Peng and Chen, 2023). Furthermore, phytopathogens release toxins to facilitate host entry, targeting the plant cell membrane, mitochondria, and chloroplasts, thereby disrupting plant cellular integrity or metabolism (Shang et al., 2016). Virulence factors in pathogenic fungi, such as phospholipases, lipases, proteases, hemolysins, phosphatases, ureases, and other hydrolases, form an effective arsenal for successful penetration, survival, and dissemination within the host (Satala et al., 2023). GO annotation of VAP-1 proteins highlights “proteolysis” as the most significantly enriched term in the “biological process” domain.

Comparative genomics analysis of the VAP-1 genome with *R. solani* genomes infecting rice, sugar beet, and tobacco reveals a closer species-level relationship between VAP-1 and the rice isolate, with a greater degree of genomic divergence observed in the isolates infecting sugar beet and tobacco. It has been demonstrated that closely related species often exhibit shared infection strategies and virulence mechanisms, including the production of mycotoxins and tissue-degrading enzymes (Aoki and O’Donnell, 1999). As pathogenic fungi adapt to different host plants and environmental conditions, they can evolve distinct pathogenic strategies. Evolutionary divergence among species can lead to variations in virulence and infection mechanisms (Talbot, 2003). Orthologous clustering of VAP-1 protein sequences with those from *R. solani* infecting rice, sugar beet, and tobacco resulted in a single large cluster comprising 4,028 proteins common to all fungal genomes, while a distinct singleton cluster of 105 proteins was unique to the VAP-1 genome. Gene Ontology (GO) analysis of these 105 VAP-1 specific proteins revealed significant enrichment in pathways related to oxalate metabolic processes, pathogenesis, polysaccharide catabolic processes, and carbohydrate catabolic processes. The oxalate metabolic process is critical for the pathogenicity of plant pathogenic fungi, contributing to infection, colonization, and tissue damage by altering the host plant’s pH, degrading cell walls, disrupting calcium signaling, and suppressing immune responses (Dutton and Evans, 1996). *R. solani* utilizes various pathogenic factors, including secondary metabolites, carbohydrate-active enzymes, secreted proteins, and effectors to facilitate infection and virulence against its host plants (Li et al., 2021). During infection, *R. solani* secretes a range of cell wall-degrading enzymes that break down complex polysaccharides in the plant cell wall, enabling the fungus to access the plant’s cellular contents and facilitate penetration and colonization of plant tissues (Xue et al., 2018). The degradation of plant cell wall polysaccharides through carbohydrate catabolism allows *R. solani* to adapt to different plant environments by utilizing various plant-derived carbohydrates (Majumdar et al., 2022). This metabolic flexibility in nutrient acquisition enhances the pathogen’s ability to infect a wide range of host plants, including rice, sugar beet, tobacco, and, as shown in our study, proso millet. Comparative genomics has significantly advanced our understanding of fungal pathogens, offering critical insights into their genetic composition, virulence factors, and mechanisms of adaptation. This knowledge is pivotal for the development of innovative disease management strategies, such as the design of targeted fungicides, biocontrol agents, and the breeding of resistant plant varieties. Despite challenges, particularly those related to genomic complexity and the evolution of resistance, comparative genomics remains an invaluable tool for addressing fungal diseases in enhancing plant health.

Secretory proteins encoded in the VAP-1 genome are classified into several functional categories, including MFS general substrate transporters, (Trans) glycosidases, P-loop containing nucleoside triphosphate hydrolases, and galactose oxidase within the central domain superfamily. The protein repertoire, particularly carbohydrate-active enzymes (CAZymes), play a pivotal role in degrading plant cell wall polymers and releasing cell wall-bound saccharides, which serve as carbon sources for the actively infecting fungus (Hage and Rosso, 2021). Among the CAZymes identified in the VAP-1 genome, glycoside hydrolases constitute the largest group, followed by auxiliary activities, polysaccharide lyases, glycosyltransferases, and carbohydrate esterases. Notably, cellulases, xylanases, and pectinases, which degrade polysaccharides, are part of the glycoside hydrolase family and may play critical roles as pathogenicity factors in the infection of wheat spikes by *Fusarium graminearum* (Wanjiru et al., 2002). Glycoside hydrolases are particularly significant as they degrade plant cell wall polysaccharides, facilitating fungal penetration and colonization (Kubicek et al., 2014). Similarly, AAs contribute to oxidative degradation of lignocellulosic components, further assisting in tissue maceration (Hemsworth et al., 2015). Auxiliary activities represent a diverse group of enzymes that support glycoside hydrolases by catalyzing the oxidation of carbohydrates, often in synergy with other enzymatic activities. This collaboration enhances the degradation of plant cell walls, facilitating fungal invasion and colonization (Levasseur et al., 2013). Additionally, glycosyltransferases (GTs), enzymes responsible for catalyzing the transfer of sugar moieties to acceptor molecules, play a crucial role in fungal pathogenesis. For example, glycosyltransferases are involved in regulating fumonisin biosynthesis and virulence in *Fusarium verticillioides*, a major global pathogen of maize, highlighting their importance in the infection process (Deng et al., 2021). The ability of *R. solani* to produce a diverse array of CAZymes indicates its efficiency in degrading plant defenses and sustaining infection.

*Rhizoctonia solani* is a polyphagous fungal pathogen that causes diseases in a wide range of crops. However, the molecular mechanisms underlying its pathogenicity, particularly the factors related to genomic complexity and evolving proteins, remain poorly understood (Francis et al., 2023). The secretomes of *R. solani* employ distinct virulence strategies, including host invasion, immune evasion, adaptation to saprophytic phases, highlighting its genomic complexity (Anderson et al., 2017). Additionally, *R. solani* secretes a diverse array of secondary metabolites, including host-selective toxins and other biologically active compounds, which play a crucial role in its virulence (Li et al., 2021).

Gene loss has been associated with host jumps in other fungi, such as the smut fungus *Melanopsichium pennsylvanicum*, which shows variations in putative secreted proteins during host shifts (Sharma et al., 2014). In our study, we observed variations in several secretome superfamily proteins of *R. solani*. Specifically, two gene families exhibited contraction in the *R. solani* strain infecting rice, while a single gene family contracted in the strain infecting sugar beet. Furthermore, we found variable numbers of base deletions in the major facilitator superfamily (MFS) proteins of *R. solani* isolates infecting proso millet, rice, and sugar beet, when compared to the tobacco isolate. Similarly, variations were noted in proteins such as glycoside hydrolase family 10, P-loop containing nucleoside triphosphate hydrolases, and galactose oxidase, all of which are key components of the pathogen’s secretory protein arsenal. These findings suggest that the observed variations may either be critical for host specificity or indicate the existence of alternative mechanisms that compensate for the functional loss of certain proteins during host infection. Gene family expansion and contraction are pivotal evolutionary mechanisms that enable pathogenic fungi to adapt, enhance stress resilience, and refine their strategies for host infection (Meng and Tian, 2023). The expansion of gene families equips the pathogen with an expanded arsenal for adaptation, facilitating increased virulence and resistance to environmental pressures (Gladieux et al., 2014). In contrast, contraction allows for more specialized and optimized functions tailored to particular environments or host interactions (Stajich, 2017). This dynamic suggests that a delicate balance between these opposing processes is essential for the evolutionary success of pathogens, influencing their capacity to persist amidst environmental fluctuations and host immune responses. Investigating these evolutionary patterns provides valuable insights into pathogen biology and can inform the development of novel strategies for disease control. In addition, a deeper understanding of the secretome and effector biology of *R. solani* is crucial to improve our ability to mitigate the impact of its multifunctional secreted effectors, which present substantial challenges to the immune responses of proso millet.

This study presents a comprehensive genomic analysis of *R. solani* infecting proso millet, laying a critical foundation for the development of targeted control strategies through detailed genome and protein sequence data. We propose a bidirectional approach that emphasizes the importance of in-depth analysis of both the host and pathogen genomes. A thorough understanding of the *R. solani* secretome will greatly enhance our ability to counteract its multifunctional effectors, which pose significant challenges to the host defenses of proso millet. Further investigation into the genes associated with pathogenicity may lead to the development of innovative management strategies, such as host-induced gene silencing to control disease or advanced molecular tools like CRISPR for the creation of disease-resistant cultivars. Nonetheless, it is crucial to conduct functional assays such as gene knockout studies to validate the identified effector proteins and CAZymes to ensure their precise role in pathogenicity.

## Conclusion

Proso millet, a key minor millet cultivated for food, feed, and fodder across Asia, is especially valued for its resilience to adverse conditions. However, its productivity is increasingly threatened by Bsb, caused by the soil-borne fungus *R. solani*. In this study, we identified the most virulent strain of *R. solani*, designated VAP-1, which infects proso millet. We sequenced and *de novo* assembled its genome with high completeness, predicting and annotating functional regions to support further functional genomic research. The VAP-1 genome construction utilized 99.55% of the quality-filtered reads, resulting in a total genome size of 47.12 Mb. The repetitive element landscape of the genome is predominantly composed of interspersed repeats, which are known to play a critical role in the rapid evolution of fungal pathogens, enhancing their adaptability and pathogenicity. Functional analysis revealed that the most enriched KEGG pathways included “Carbohydrate metabolism,” “Translation,” “Signal transduction,” and “Transport and catabolism,” while the Gene Ontology (GO) terms “Proteolysis,” “Membrane,” and “ATP binding” showed the highest enrichment, highlighting key processes involved in the virulence and metabolic flexibility of the pathogen.

The secretory protein repertoire of the VAP-1 genome from *R. solani* includes key proteins from the Major Facilitator Superfamily (MFS) transporters, (Trans) glycosidases, P-loop containing nucleoside triphosphate hydrolases, and galactose oxidase within the central domain superfamily. These proteins are essential for enabling fungi to invade plant tissues, acquire nutrients, and evade host defenses, all of which are crucial for the pathogen’s success in infecting and colonizing plant hosts. Among these, glycoside hydrolases, which represent the largest class of CAZymes in the VAP-1 genome, are particularly important in promoting host colonization.

Comparative genomic analysis between VAP-1 and other *R. solani* strains infecting the Poaceae (e.g., rice) and non-Poaceae (e.g., sugar beet and tobacco) families reveals that VAP-1 clusters closely with strains infecting rice at the species level, but shows greater divergence from those infecting sugar beet and tobacco in terms of genomic similarity. Further examination of the variations in the secretory proteins highlighted multiple base deletions in MFS proteins across strains infecting proso millet, rice, and sugar beet. Additionally, variations were observed in glycoside hydrolase family 10, P-loop containing nucleoside triphosphate hydrolases, and galactose oxidase, which are all crucial components of the pathogen’s secretory protein arsenal.

These findings offer valuable insights into the genetic composition and potential virulence factors of *R. solani* strain VAP-1. The observed variations suggest that this strain may employ distinct molecular mechanisms or unique strategies to successfully infect proso millet, contributing to a deeper understanding of its pathogenesis. A thorough investigation of the genes associated with pathogenicity has the potential to uncover innovative strategies for disease management, especially in case of the secretome proteins. Understanding virulence factors paves the way for targeted disease control strategies. The identified secreted proteins provide potential targets for host-induced gene silencing (HIGS), to disrupt fungal pathogenicity genes. Similarly, CAZymes can be inhibited to suppress fungal enzymatic activity and impede infection. It is essential to conduct functional assays to validate the roles of these effector proteins and carbohydrate-active enzymes (CAZymes), as understanding their precise functions is critical for designing targeted and effective control measures. Bio-control agents like *Trichoderma* spp. and *Bacillus* spp. offer another strategy, secreting antagonistic metabolites that degrade fungal cell walls or inhibit virulence factors, reducing disease incidence. Additionally, the genomic insights gained from this study also hold promise for breeding disease-resistant proso millet varieties. Effector proteins secreted by *R. solani* interact with host susceptibility genes, making them ideal targets for genome-editing approaches such as CRISPR-Cas9. By knocking out or modifying host susceptibility genes, millet varieties can be engineered to exhibit increased resistance to *R. solani*.

## Data Availability

The whole-genome shotgun sequencing of *Rhizoctonia solani* strain VAP-1 has been deposited in the NCBI SRA database under BioProject ID PRJNA1132789 and BioSample ID SAMN42358610. The corresponding whole-genome assembly of *R. solani* (VAP-1 strain) is available in the NCBI repository under Genome submission ID SUB14592034 and accession number JBFDVA000000000.
