# Supplementary material for: Unraveling the pathogenomics of Rhizoctonia solani infecting proso millet (Panicum miliaceum L.): genomic perspective on ruthless virulence and adaptive evolution
Source: Front Microbiol. 2025 Mar 7;16:1557991. doi: 10.3389/fmicb.2025.1557991 (PMC11925929; doi:10.3389/fmicb.2025.1557991)
Supplement: Supplementary file 1 [file Data_Sheet_1.docx]

**Supplementary Table 1:** Characteristics of hyphal and sclerotial parameters for various *Rhizoctonia solani* isolates analyzed in the current study.

| **Sl. No.** | **Isolate** | **Sclerotia pattern of arrangement** | **Sclerotia**  **Texture** | **Diameter of**  **sclerotia (mm)** | **Sclerotia number** | **Hyphal width (μm)** |
| --- | --- | --- | --- | --- | --- | --- |
| 1 | BOD-01 | Central | Rough | 1.67 ± 0.07 | 15 | 5.02 ± 0.20 |
| 2 | SOD-02 | Peripheral ring | Rough | 0.93 ± 0.05 | 27 | 7.33 ± 0.33 |
| 3 | JCG-01 | Central | Rough | 1.47 ± 0.04 | 13 | 7.29 ± 0.65 |
| 4 | RCG-02 | Central as well as sub-central | Smooth | 1.20 ± 0.08 | 32 | 7.42 ± 0.17 |
| 5 | VAP-01 | Central as well as sub-central | Smooth | 1.66 ± 0.04 | 21 | 5.87± 0.01 |
| 6 | HAP-02 | Central | Rough | 1.79 ± 0.01 | 29 | 5.28± 0.05 |

**Supplementary Table 2**: VAP-1 genome’s raw read and filtered read statistics

| **VAP1 (Read Quality)** | **Total Sequences** | **Sequence length** | **Percent (%) of sequences** | **GC%** |
| --- | --- | --- | --- | --- |
| Raw Reads | 7276805 | 154 | 100.00 | 47 |
| Filtered Reads | 7275429 | 18-144 | 99.98 | 47 |

**Supplementary Table 3:** VAP-1 genome assembly statistics generated by QUAST for de novo, genome-guided correction, scaffolding, repetitive contig filtering, and retention of contigs ≥500 bp, compared to AG-1 IA strain genome

| **Assembly metric** | ***de novo* assembly** | **RagTag assembly** | **Funannotate (final VAP1 assembly)** | **GCF_016906535.1 (AG-1 IA)** |
| --- | --- | --- | --- | --- |
| Assembly size (bp) | 58478347 | 61432747 | 47124650 | 40856322 |
| Total contigs | 114204 | 84669 | 6096 | 17 |
| Largest contig (bp) | 68865 | 3918178 | 3918178 | 3756861 |
| N50 (bp) | 2988 | 2122470 | 2122470 | 2303118 |
| N90 (bp) | 716 | 2398 | 2398 | 2094219 |
| L50 | 3177 | 8 | 8 | 7 |
| L90 | 15568 | 1207 | 1207 | 14 |
| # N's per 100 kbp | 472.48 | 6674.1 | 6674.1 | 0.00 |

**Supplementary Figures:**


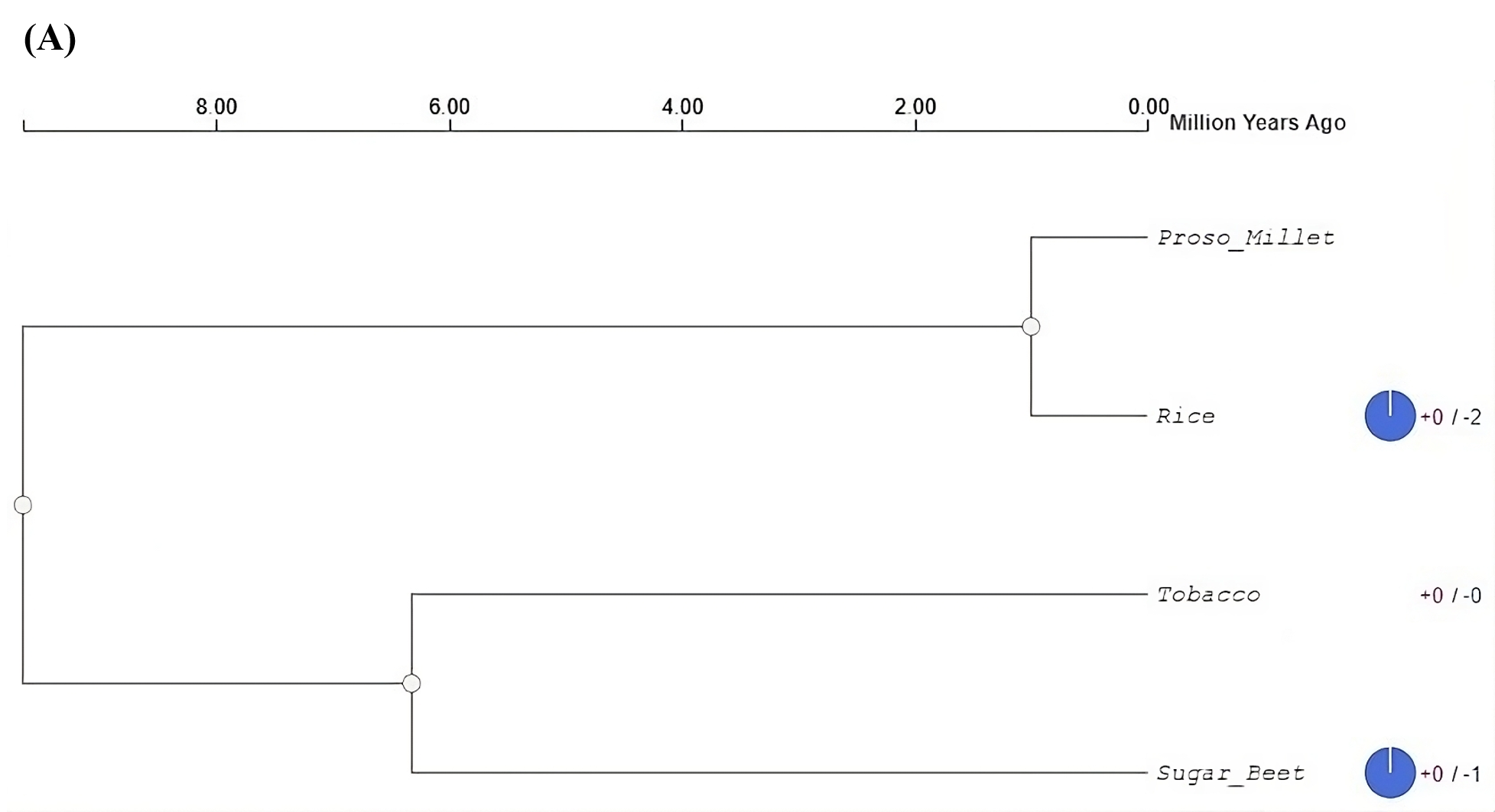

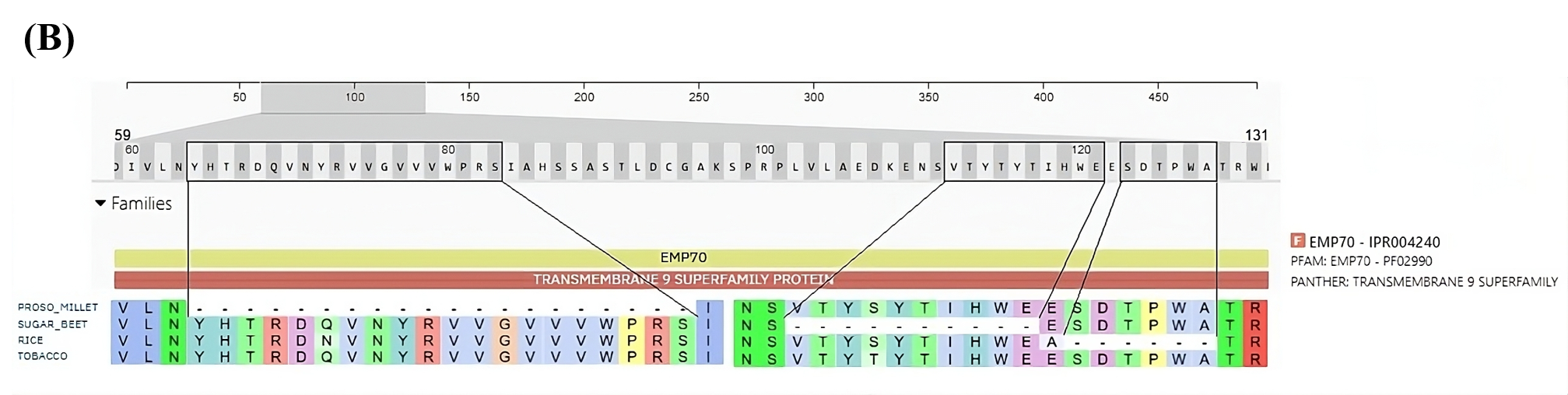

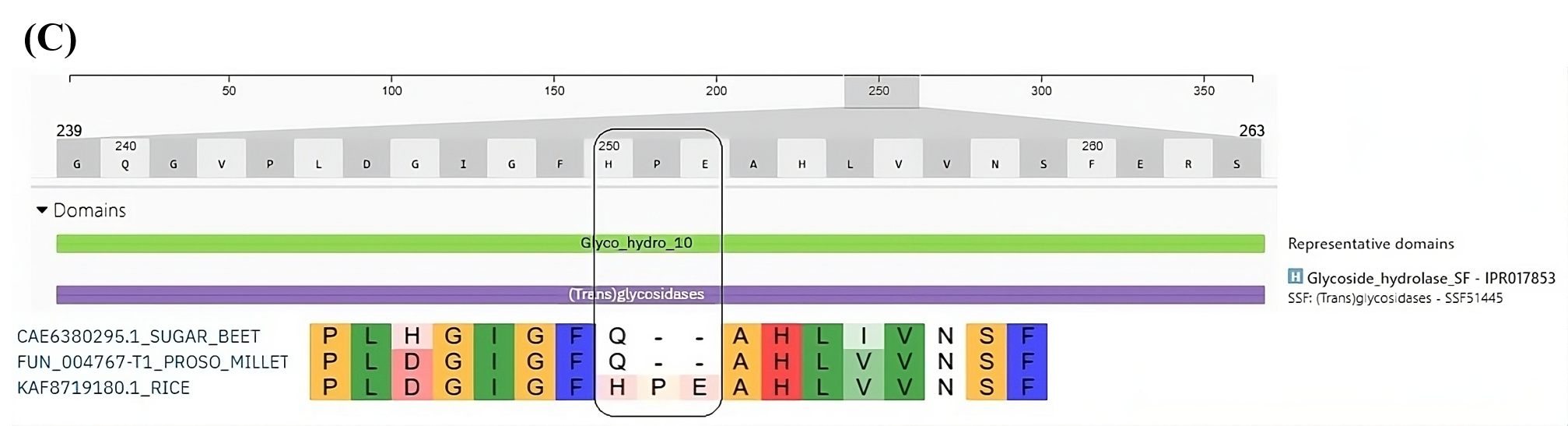


**Supplementary Figure S1**: Evolution and Variation in Secretome Proteins **(A)** Phylogenetic tree illustrating the expansion and contraction of gene families across *R. solani* genomes. **(B)** Base deletions in the Major Facilitator Superfamily protein across *R. solani* genomes. **(C)** Variations, including single base mutations and consecutive two-base deletions, in (Trans) glycosidases superfamily enzymes in *R. solani* infecting sugar beet, Proso millet, and rice.


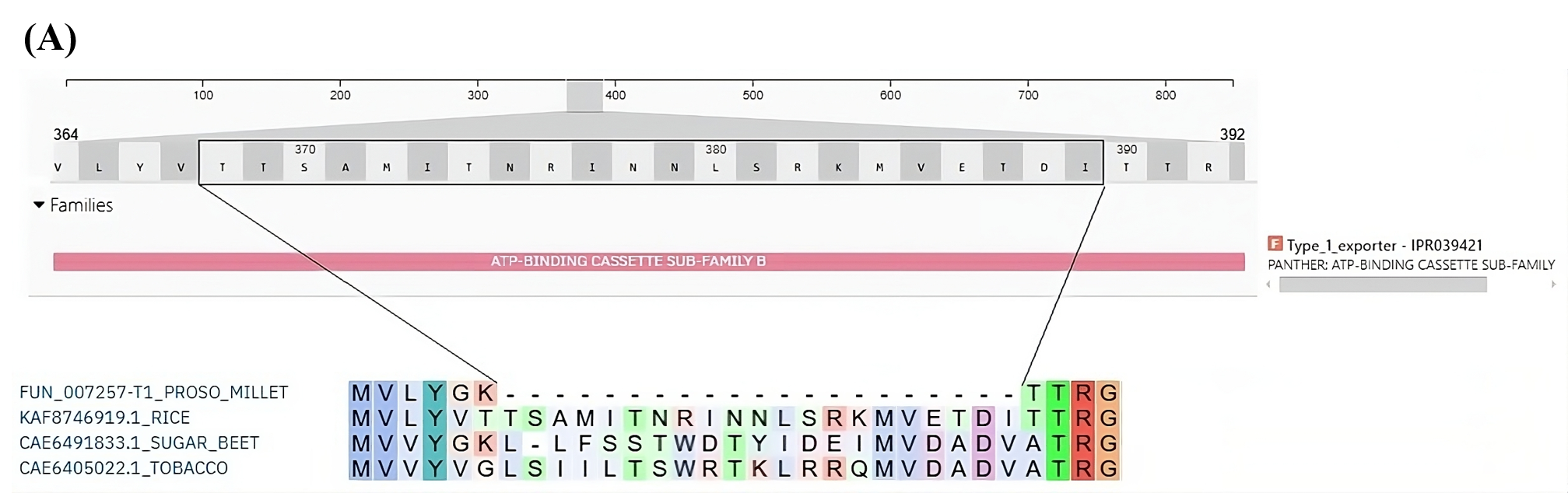

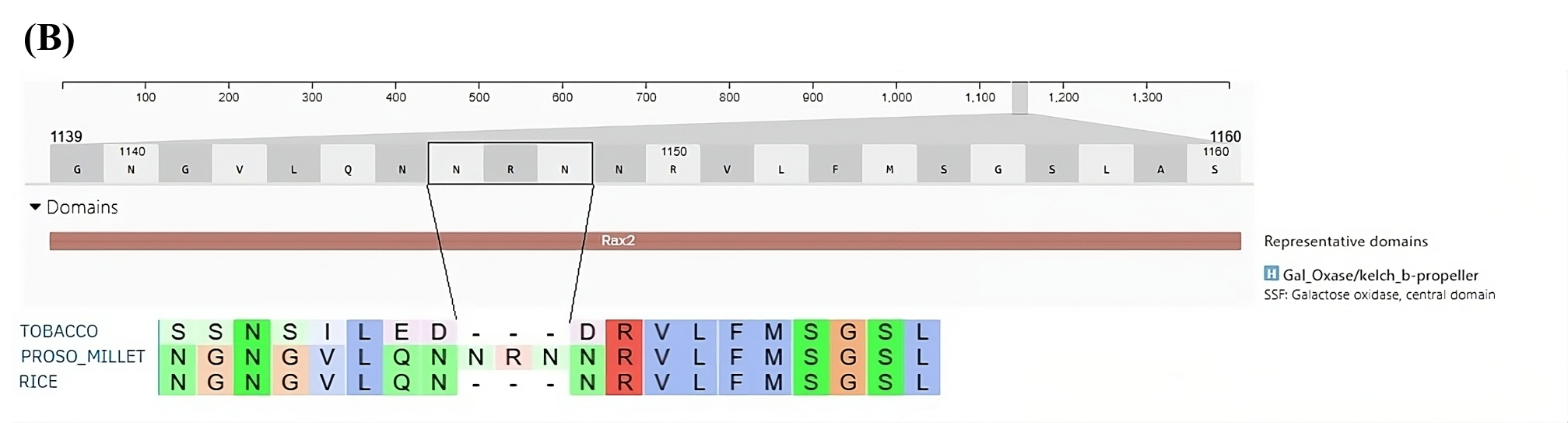


**Supplementary Figure S2:** Variation in Secretome Proteins **(A)** Base deletions in ATP-binding cassette subfamily B proteins across *R. solani* genomes. **(B)** 3-base deletion in the RAX2 domain of galactose oxidase proteins from *R. solani* infecting tobacco, proso millet, and rice
